# Therapeutic Approaches Targeting Proteostasis in Kidney Disease and Fibrosis

**DOI:** 10.3390/ijms22168674

**Published:** 2021-08-12

**Authors:** Jia-Huang Chen, Chia-Hsien Wu, Chih-Kang Chiang

**Affiliations:** 1 Graduate Institute of Toxicology, College of Medicine, National Taiwan University, Taipei 100233, Taiwan; f04447010@ntu.edu.tw (J.-H.C.); r03447008@ntu.edu.tw (C.-H.W.); 2 Department of Physiology of Visceral Function and Body Fluid, Nagasaki University Graduate School of Biomedical Sciences, Nagasaki 852-8523, Japan; 3 Department of Integrated Diagnostics & Therapeutics, National Taiwan University Hospital, Taipei 100225, Taiwan; 4 Center for Biotechnology, National Taiwan University, Taipei 10672, Taiwan

**Keywords:** unfolded protein responses, endoplasmic reticulum stress, fibrosis, kidney, proteostasis

## Abstract

Pathological insults usually disturb the folding capacity of cellular proteins and lead to the accumulation of misfolded proteins in the endoplasmic reticulum (ER), which leads to so-called “ER stress”. Increasing evidence indicates that ER stress acts as a trigger factor for the development and progression of many kidney diseases. The unfolded protein responses (UPRs), a set of molecular signals that resume proteostasis under ER stress, are thought to restore the adaptive process in chronic kidney disease (CKD) and renal fibrosis. Furthermore, the idea of targeting UPRs for CKD treatment has been well discussed in the past decade. This review summarizes the up-to-date literature regarding studies on the relationship between the UPRs, systemic fibrosis, and renal diseases. We also address the potential therapeutic possibilities of renal diseases based on the modulation of UPRs and ER proteostasis. Finally, we list some of the current UPR modulators and their therapeutic potentials.

## 1. Introduction

Kidney diseases have recently received considerable attention because the renal function is vulnerable to pathogenic insults, including inflammation, hypoxia, hypertension, and aging. Once acute kidney injury (AKI) occurs, the event frequently leads to the progression of chronic kidney disease (CKD) despite a transient recovery from AKI [1]. Renal tubulointerstitial fibrosis is a common pathway of advanced CKD, which is associated with vasoconstriction, capillary obliteration caused by fibrotic expansion, and finally the formation of a hypoxic microenvironment that worsens renal function [2,3].

There is accumulating evidence indicating that the disruption of endoplasmic reticulum (ER) homeostasis is involved in various pathological processes, including cancer, metabolic diseases, diabetes, neurodegenerative disorders, and liver dysfunction [4]. The unfolded protein responses (UPRs) signaling activated by the ER stress participates in the progression of AKI and CKD [5]. In addition, ER stress is also involved in the progression of organ fibrosis, including those of the kidney, liver, and lung [6]. Because of the limited therapeutic options for the retarding of CKD progression, the modulation of UPRs signaling has become an attractive target for drug discovery [7]. In this mini-review, we discuss the role of UPRs in renal diseases and renal fibrosis, highlight the therapeutic potentials of the modulation of UPRs and the proteostasis of ER, and, in particular, emphasize the role of inositol-requiring protein 1-X-box-binding protein 1 (IRE1-XBP1) signaling.

## 2. Fundamental Roles of ER Stress and Unfolded Protein Responses

The ER is the organelle where most secretory and transmembrane proteins are synthesized, modified, and folded into their correct conformations. Since the ER plays such an essential role in the maintaining of proteostasis, it must optimally control the quality of protein folding [8]. However, the folding capacity of the ER is susceptible to environmental stress, such as inflammation, oxidative stress, infection, or the deprivation of nutrients, which leads to the abundant accumulation of misfolded or unfolded protein in the ER, which is hence termed the “ER stress” [9]. Restoring the proteostasis of the ER will initiate the signaling of UPRs, thereby reducing protein load, increasing folding ability, or triggering cell apoptosis for as long as overwhelming ER stress persists [8]. Three ER transmembrane proteins, including IRE1, pancreatic eukaryotic translation initiation factor 2-alpha kinase (PKR-like protein kinase, PERK), and activating transcription factor 6 (ATF6), mediate the transduction of UPRs signaling. In normal proteostasis, the immunoglobulin heavy-chain binding protein (BiP) will conjugate with these three transmembrane proteins, and no UPRs cascade will be initiated. When ER stress occurs, BiP acts as an endogenous chaperon that binds to misfolded proteins, and therefore, detaches from the UPRs initiators, resulting in the activation of UPRs signaling [10].

Upon ER stress, IRE1 is activated after the dissociation of BiP, which leads to the conformational change of IRE1. This promotes its dimerization and trans-autophosphorylation, followed by activation of the kinase and RNase domains [11,12]. XBP1 RNA is spliced by unconventional endonuclease, causing a frameshift and generating spliced XBP1 (XBP1s), which possesses a completely different function from its intact form (XBP1u) [13]. XBP1s acts as a transcription activator, upregulating genes that encode ER-associated degradation (ERAD) and chaperone [14,15]. However, the overwhelming level of ER stress induces the activation of the kinase domain, and then recruits tumor necrosis factor receptor (TNFR)-associated factor-2 (TRAF2), before activating the signaling of c-Jun amino-terminal kinases (JNK), which are pro-apoptosis mediators [16]. In addition, hyper-activation of IRE1α is reported to activate regulated IRE1α-dependent degradation (RIDD) and digest a subset of mRNAs that encode secretory proteins [17,18].

The manner of activation of PERK is similar to the way in which IRE is activated. After dimerization and trans-autophosphorylation, PERK suppresses global protein translation by phosphorylating the α subunit of eukaryotic translation initiation factor-2 (eIF2α) [19]. However, ATF4, a critical transcriptional regulator in the PERK-eIF2α pathway, is upregulated by ribosomal skipping [20]. Following the activation of downstream stress-induced redox proteins, C/EBP-homologous protein (CHOP) [21], which is well recognized as a pro-apoptotic protein that mediates UPR-related cell death [22], directly activates both growth arrest and DNA damage-inducible protein-34 (GADD34) [23], the protein that dephosphorylates the phospho-eIF2α in cells under ER stress and helps cells to recover from translational inhibition [23].

Activated ATF6 translocates from the ER to the Golgi apparatus, and is cleaved by site-1 and site-2 proteases in the Golgi apparatus [24,25], thereby generating the cleaved fragment of ATF6 (cATF6), and then cATF6 enters the nucleus to activate the genes that enhance protein folding, including BiP, GRP94, calreticulin, protein disulfide isomerase (PDI), and XBP1 [26,27,28,29].

The three-branch axis orchestrates the process of UPRs, and their interdependency in terms of regulation is well documented. For instance, the downstream target genes of ATF6 can be compensated by XBP1 during acute silence of ATF6 [30]. Inhibition of PERK leads to the compensatory activation of XBP1s, and the inhibition of IRE1α contributes to the sustained activation of PERK and CHOP [31]. In addition, hyperactivation of IRE1α is found in the XBP1 deletion hepatocyte [32]. Recent evidence demonstrated that cell fate is determined by elaborate ER compensation during ER stress.

## 3. Homeostatic Role of Proteolysis through Adaptive UPRs Activation in Disease Progression

ERAD plays a pivotal role in the removal of misfolded protein to maintain the homeostasis and cell survival of ER. The process of ERAD is initiated by unfolded substrate recruitment, assisted by chaperones such as Bip and the ER degradation-enhancing alpha-mannosidase-like protein (EDEM) protein family. The tagged protein is eventually degraded by the 26S proteasome and resolves ER stress [33,34]. However, it may shut down the capacity of ERAD and activate apoptotic ER stress during the overloading of misfolded/unfolded proteins, as well as during oxidative stress or heat shock states [35]. Various ERAD-deficient mouse models developed organ dysfunction, including enteritis, obesity, and glucose intolerance [36,37,38]. In addition, ERAD inhibition was identified as a therapeutic target in cancer treatment [39,40]. Furthermore, the enhancement of ERAD through the overexpression of XBP1s reduced amyloid β-peptide accumulation in an Alzheimer’s disease model [41,42].

## 4. ER Stress-Mediated Autophagy and Proteostasis

There is strong evidence supporting the crosstalk between ER stress and autophagy [43,44,45]. Autophagy is triggered by a mechanistic rapamycin (mTOR) inhibition target and involves sequential steps, including the initiation of phagophore, which begins with Unc-51-like autophagy activating the complex formation and nucleation of kinase 1 (ULK1). Then, the autophagosome membrane elongation is assisted by the conjugation of autophagy-related protein (ATG) to LC3 phosphatidylethanolamine (PE) and the lysosome, and finally, proteolytic degradation is initiated [46].

The interplay between ER stress and autophagy has been frequently mentioned. For example, the accumulation of cytosol calcium from ER will activate UPRs, followed by the inhibition of mTOR and the induction of autophagy [47]. Margariti et al. and Ogata et al. demonstrated that autophagy transcriptionally induced the activation of beclin-1 via the IRE1/XBP1s and IRE1/JNK axis [48,49]. B’chir et al. elegantly showed that induction of PERK/eIF2α/ATF4 axis is essential for ATGs genes expression [50]. Furthermore, Fang et al. and Qi et al. showed that the chemical chaperone 4-PBA and TUDCA can attenuate STZ and obesity-induced diabetic nephropathy, extracellular matrix deposition, and autophagy in an ER-stress-dependent manner [51,52]. All of the above-mentioned reports support the connection between ER stress and autophagy.

## 5. UPRs and Systemic Fibrosis Progression

Many studies proposed the relationship between UPRs and organ fibrosis [6,53]. Familial interstitial pneumonia (FIP), a class of interstitial pneumonitis that may be caused by the genetic mutation in surfactant protein C (SPC) [54], suggests a potential connection between organ fibrosis and ER stress. SPC is secreted by type II alveolar epithelial cells in order to maintain alveolar distensibility. In vitro studies revealed that the mutation of SPC in the carboxyl domain leads to its accumulation, in a misfolded form, in the ER lumen [55]; furthermore, tissue samples from FIP patients with SPC mutation showed prominent expression of BiP, and XBP1 expression co-localized with fibrotic areas [56].

In murine models of cardiac fibrosis, subcutaneous injection of isoproterenol and angiotensin II-induced fibrosis activated UPR signaling and upregulated the pro-apoptotic expression of CHOP. In these models, the severity of fibrosis was attenuated through the administration of chemical chaperone 4-phenylbutyric acid (4-PBA) [57,58].

## 6. UPRs in Renal Disease and Fibrosis

Renal fibrosis is the final common pathway of CKD and end-stage renal disease (ESRD) [59], which results from the loss of parenchymal due to the occurrence of natural senescence, diabetes, or acute kidney insults. It has been well documented that AKI is recognized as a cause of long-term risk of CKD or ESRD and maladaptive repair of kidney injury, leading to renal fibrosis and the transition from AKI to CKD [60,61]. Myofibroblasts play a critical role in the inducing of excessive deposition of the extracellular matrix, which contributes to renal fibrosis. Myofibroblasts can originate from the activation of renal interstitial fibroblasts, perivascular fibroblasts, pericytes, and bone marrow-derived mesenchymal cells, as well as the transition of endothelial cells or tubular epithelial cells [62,63,64,65,66]. Recent studies revealed that perivascular fibroblasts and pericytes, but not injured tubular epithelial cells, transdifferentiate into myofibroblasts and contribute to fibrosis in renal fibrosis animal models. However, an emerging concept is that kidney damage caused by AKI or unresolved injuries leads to prolonged cell arrest in the cell cycle G2/M phase and leads to the appearance of profibrotic and proinflammatory features in tubule cells. Profibrogenic growth factors and inflammatory cytokines secreted from injured tubules can stimulate the proliferation of fibroblasts and the production of extracellular matrix, and eventually contribute to progressive renal fibrosis. In addition, many publications also revealed that UPRs signaling is involved in the progression of renal diseases, which is triggered by hypoxia, oxidative stress, inflammation, high glucose, and functional genetic deficiency of the glomerular protein [67].

### 6.1. Disturbance of UPR Contributes to AKI-to-CKD Transition

Renal ischemia-reperfusion injury is a common cause of AKI that results in hypoxia, and ER stress is well recognized as the initial response to ischemia-reperfusion injury [68,69]. A calcitonin/calcitonin gene-related peptide, namely intermedin, ameliorates renal ischemia-reperfusion injury by inhibiting ER stress-mediated apoptotic signaling, for example, the expression of CHOP and caspase-12 [70]. The unilateral ureteral obstruction (UUO) model activated all three effectors of the UPR signaling during the development of renal fibrosis. The pro-apoptotic signals, such as CHOP, caspase-12, JNK, and Bax, were also increased [65]. Furthermore, Fan and Xiao et al. showed that the dysregulation of UPRs was correlated to the severity of the progression from AKI to CKD in humans, with upregulated expression of Bip, p-PERK, and CHOP and reduced expression of XBP1s in patients with progressive AKI renal biopsy [71]. Jao et al. revealed that the dysregulation of UPRs induced the accumulation of lipids, as well as renal fibrosis [72]. The renal fibrosis induced in the UIRI mice model coincides with the accumulation of lipids and the activation of ATF6 in tubular epithelial cells. Furthermore, ATF6 knockout mice demonstrated less tubulointerstitial fibrosis and lipid accumulation through the activation of PPARα.

The production of reactive oxygen species (ROS) during renal ischemia-reperfusion injury also causes the pathogenesis of CKD progression [73]. Antioxidant therapy for CKD patients showed significant benefits, including attenuation of the risk of ESRD development, reduced serum creatinine levels, and improved creatinine clearance [74]. The production of ROS interferes with cellular redox-dependent metabolism and protein-folding capacity, resulting in the accumulation of misfolded proteins in the ER [75]. The antioxidative effects of UPRs were also found during the stimulation of cells against ROS [76]. Nuclear factor E2-related factor 2 (Nrf2) is an antioxidative transcription regulator that resists oxidative stress through the activation of antioxidative genes, such as catalase, heme oxygenase-1 (HO-1), and superoxide dismutase [77]. In terms of the connection between Nrf2 and three branches of UPRs initiators, it was demonstrated that Nrf2 is the downstream target of the ATF6 [78], IRE1/JNK [76], and PERK pathways [79]. The PERK-Nrf2 pathway, for example, plays an essential role in the maintenance of redox homeostasis, as shown by the fact that a deficiency of PERK leads to the accumulation of ROS in cells [80]. Cadmium induces kidney injury through the generation of ROS and leads to ER stress-mediated apoptosis [81]. This evidence suggests that ER stress is an important pathogenic mediator of renal diseases.

### 6.2. Dysregulation of UPR Mediates Renal Fibrosis in Diabetic Nephropathy and Podocyte Defect Mice Model

Diabetic nephropathy (DN) accounts for up to 40% of incident ESRD [82]. In the streptozotocin (STZ)-induced DN model, the upregulation of BiP and CHOP, and the activation of PERK signaling, are observed in 22-month-old mice with DN accompanied by tubulointerstitial fibrosis and extensive inflammatory cell infiltration [83]. The renal renin-angiotensin system (RAS) is well known for governing blood pressure and the homeostasis of target organs. In the cascade of RAS, angiotensin II is the key regulator that contributes to vasoconstriction. In addition, the proinflammatory and profibrogenic effects of angiotensin II are widely discussed in the progression of CKD [84]. Therefore, angiotensin-converting enzyme inhibitor (ACEI) or angiotensin receptor blocker (ARB) are the standard treatments for hypertension or heart failure patients. Evidence showed that ACEIs reduce the apoptosis of renal tubular cells and suppress the signaling of UPRs, as demonstrated by, for example, the activation of phospho-eIF2α and phospho-PERK in STZ-induced diabetic rat models [85]. Human kidney biopsy samples demonstrated that diabetic nephropathy had higher expression of BiP, XBP1, and CHOP, which is consistent with in vitro findings. In addition, albumin and high glucose administration induced the activation of ER stress in human and rodent renal tubules [86]. Furthermore, the deletion of podocyte-specific IRE1a and XBP1s in mice led to more severe cases of albuminuria, glomerular basement membrane thickening, and ER stress induction [87,88].

## 7. Therapeutic Strategies: Targeting the IRE1-XBP1 and PERK-eIF2α Axis

### 7.1. IRE1-XBP1 Axis of UPRs

**Figure 1:**
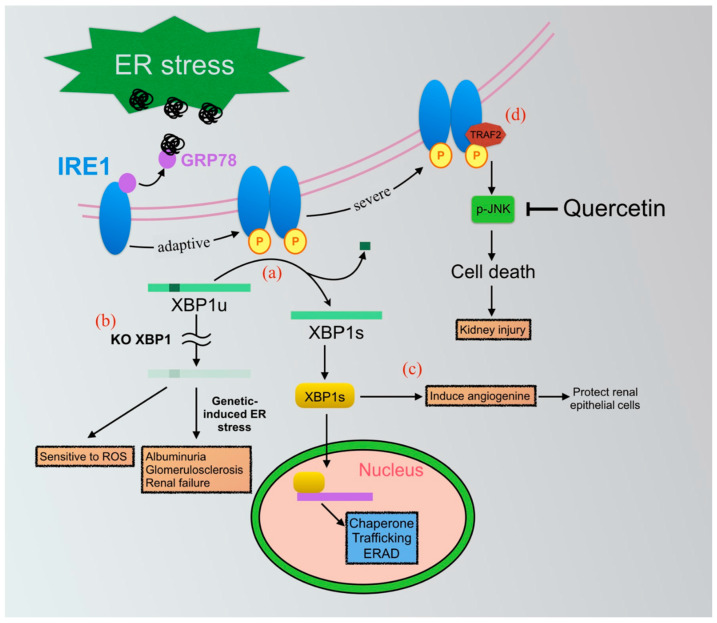
The IRE1-XBP1-related UPRs pathways played pathological and therapeutic roles in kidney disease. (**a**) Unfolded proteins bind to BiP and allow the activation of IRE1 to occur through its dimerization and trans-autophosphorylation. IRE1 phosphorylation as indicated p in the yellow cycle. Activated IRE1 splices the mRNA of XBP1u to form spliced XBP1 (XBP1s) mRNA, which encodes XBP1s protein then translocates to the nucleus, inducing genes that are thought to be generally cytoprotective, such as chaperones, trafficking associators, ERAD-related proteins. (**b**) Evidence shows that XBP1 knockout makes cells sensitive to oxidative stress, an important insult to kidney injury. Combining the XBP1 knockout model with genetically induced ER stress in the kidney leads to severe albuminuria, glomerulosclerosis, and renal failure. (**c**) XBP1s could directly induce angiogenin (ANG) expression in renal epithelial cells, which has been proven to protect cells and attenuate adverse effects caused by ER stress. (**d**) Severe ER stress triggers cell death through the IRE1-TRAF2-JNK pathway in many kinds of kidney diseases. Quercetin, a natural flavonoid, has been demonstrated to inhibit the IRE1-TRAF2-JNK pathway in unilateral ureteral obstruction (UUO), diabetic nephropathy, and cadmium-induced kidney injury. It might be a strong therapeutic option for the targeting of UPRs.

The XBP1 arm of the UPRs is generally recognized as cytoprotective. As mentioned above, the three UPRs branches are all activated in the UUO model [65]. In this model, XBP1s has been downregulated during the development of renal fibrosis, which ARB could reverse. Somlo and colleagues reported that site-specific deficiency of XBP1 in podocytes resulted in severe albuminuria, glomerulosclerosis, and kidney fibrosis in a Sec63 and XBP1 double knockout model [89,90]. In this first part of the study, the authors demonstrated the accumulation of unfolded proteins without proteinuria or pathological features in podocyte-specific Sec63 or XBP1s single knockout mice. However, podocyte-specific Sec63 and XBP1s double-knockout mice developed defects in the integrity of the glomerular filtration barrier and progressive tubulointerstitial fibrosis in association with the loss of podocytes, which occurred through the activation of the JNK-apoptotic pathway in 2-month-old mice. Moreover, the re-expression of XBP1s in vivo completely rescues chronic tubulointerstitial kidney injury in XBP1 and Sec63 double knockout mice. Madhusudhan et al. also revealed an essential role of XBP1 in DN. They demonstrated that sXBP1 lies downstream of insulin signaling, and attenuates insulin signaling in podocytes through the genetic ablation of the insulin receptor or the regulatory subunits phosphatidylinositol 3-kinase (PI3K) p85α or p85β, which impair sXBP1 nuclear translocation and exacerbate DN [87] (Figure 1).

Oxidative stress is also a potential contributor to renal fibrosis [91]. Liu and colleagues showed that mouse embryonic fibroblasts with a genetic deficiency of XBP1 are more sensitive to H_2_O_2_-induced apoptosis. The anti-oxidative properties of XBP1 resulted from the transcriptional upregulation of catalase that occurred through binding to its promoter region [92]. Another group also showed that XBP1 attenuated disturbed flow-induced oxidative stress through the upregulation of heme oxygenase 1 (HO-1) in endothelial cells [93]. Angiogenin (ANG) is a ribonuclease that promotes the adaptation of tissue to injury. By promoting the cleavage of tRNA, ANG plays a physiologically relevant, ER stress-mediated, adaptive role in the translational control of kidney injuries, in a IRE1-XBP1-dependent manner [94,95] (Figure 1). Inflammation also plays a critical role in the development of renal fibrosis. In a recent study, ER stress preconditioning could attenuate LDL-induced inflammation in human mesangial cells. Its underlying mechanism is mainly through the induction of XBP1 followed by the blocking of the IRE1α/IKK/NF-κB-mediated inflammatory response [94]. Collectively, modulation of the IRE1-XBP1 axis might be a favorable therapeutic target among ER stress-mediated injuries.

#### 7.1.1. IRE1-XBP1-Mediated Inflammasome in Renal Fibrosis

Kidney inflammation is considered to be a pathogenic factor that triggers CKD progression [96]. Prolonged inflammation resulting from mononuclear infiltration or the deposition of immunogens leads to the recruitment of fibrocytes, and consequently, renal fibrosis [97]. NOD-, LRR- and pyrin domain-containing protein 3 (NLRP3), a protein oligomerization that forms inflammasomes, is involved in the cleavage of pro-IL-1β and pro-IL-18, which contribute to renal inflammation and fibrosis in various non-diabetic kidney diseases [98,99,100,101]. NLRP3 knockout ameliorates tubular injury, inflammation, and fibrosis in the UUO kidney. Notably, the hyperactivation of IRE1α results in the degradation of miR-17 following the activation of the NLRP3 inflammasomes. By blocking the activation of XBP1s by STF-083010, the RNA endonuclease inhibitor attenuates the secretion of IL-1β during treatment with thapsigargin [102]. Therefore, the ER stress-mediated activation of NLRP3 inflammasome can be specifically attenuated by the activation of IRE1-XBP1s.

#### 7.1.2. Application of Small Molecular Compounds Targeting the IRE1-XBP1 Pathway

##### Kinase-Inhibiting RNase Attenuators (KIRA)

Hyperactivation of IRE1 may trigger the RIDD and lead to cell apoptosis. Recently, Dr. FR Papa has developed a KIRA that allosterically inhibits IRE1α kinase activity by breaking oligomers. KIRA prevents hyper-oligomerization of IRE1 and maintains the RNA endonuclease of IRE1, which allows XBP1 to be spliced properly. It successfully protected pancreatic β cells and retinal cells from ER stress-induced injury in vivo and in vitro [103]. It might be a vital molecule under research in the future; however, more tests are needed in different disease models.

##### Quercetin

Quercetin is one of the most abundant flavonoids with antioxidative properties in the human diet. Recent studies demonstrated its ability in the activation of IRE1 RNase and the inhibition of IRE1-JNK signaling [104,105,106]. Previous research studies showed that quercetin has a renoprotection effect in many types of kidney injuries, including DN [107], cadmium toxicity [108], and UUO kidney (Figure 1) [109]. Furthermore, in vitro investigation showed that the anti-fibrotic effect of quercetin is mediated through the attenuation of TGF-β1-induced collagen I and α-SMA expression in normal rat kidney fibroblast cells (NRK-49F) [110] and UUO kidney [111]. In addition, another research study demonstrated that quercetin protects glomerular endothelial cells from asymmetric dimethylarginine (ADMA)-induced apoptosis through PERK- and IRE1-associated and TGF-β-enhanced pathways [112] (Table 1).

##### XBP1 Agonists

Although the hyperactivation of IRE1-induced renal inflammation and fibrosis were found, the therapeutic effect of the IRE1 RNase targeted molecule, XBP1s, was proved by Lian Qiu et al. in a mouse model of ulcerative colitis (UC) or inflammatory bowel disease (IBD). IBD is a chronic intestinal inflammatory disease that revealed hypomorphic variants of XBP1 as a susceptibility factor that leads to IBD [113]. The XBP1 agonists, HLJ2 and (±)-8-ADC, are monomeric compounds that are extracted and modified from Ranunculaceae and Papaveraceae plant families [114]. These XBP1 agonists exert an anti-inflammatory effect toward dextran sulfate sodium-induced colitis through the inhibition of NF-κB. Furthermore, HLJ2 demonstrated an anti-epithelial mesenchymal transition (EMT) effect under TGF-β1 stimulation [115,116]. These findings suggest the promising therapeutic value of XBP1 agonists in inflammatory disease and organ fibrosis (Table 1).

### 7.2. PERK-eIF2α Axis

#### 7.2.1. Application of Salubrinal

Salubrinal, a selective inhibitor of GADD34-phosphatase-1 (PP1), which prevents dephosphorylation of eIF2α, was shown to protect cells from ER stress-induced apoptosis [117]. Cadmium increased the generation of ROS with subsequent induction of ER stress in a cultured renal proximal tubular cell line [118,119]. The administration of Salubrinal attenuated the cadmium-induced expression of BiP and CHOP, and the activation of cell death signaling of JNK, in HK-2 cells [120]. Salubrinal also ameliorates podocyte damage caused by hyperglycemia, as well as damage induced by other xenotoxicant agents such as arsenic, paraquat, cyclosporine, and cisplatin [51,121]. However, our previous animal study of the effects of salubrinal on cisplatin-induced renal cell damage showed a different conclusion from that obtained through in vitro studies. We found that salubrinal enhanced cisplatin-induced nephrotoxicity in mice, and this was accompanied by the activation of ATF4 and CHOP, and the cleavage of caspases-12, 9, and 3 [64]. Since salubrinal itself did not trigger renal cell injury in mice, the acceptable reason for this discrepancy in the results might be the hyperactivation of eIF2α and the enhanced cisplatin-triggered oxidative stress in the kidney.

More recently, salubrinal was shown to prevent the suppression of HK-2 cell proliferation by indoxyl sulfate [122]. In addition to salubrinal, other PERK signaling modulators including Guanabenz and GSK2606414 were investigated. Guanabenz, the α2-adrenergic receptor agonist that was originally used to treat hypertension, could prevent the dephosphorylation of eIF2α by GADD34 through competitive binding to PP1c [123]. GSK2606414 inhibits the activation of PERK by binding to the active site of the PERK kinase domain, thereby repressing tumor growth in several mouse xenograft models [124] (Table 1).

#### 7.2.2. CHOP Is the Most Potential Therapeutic Target

It is well recognized that CHOP expression is involved in many diseases, and the modulation of CHOP expression might be the most potent target to retard the progression of renal disease and fibrosis. In the ischemia-reperfusion injury model, CHOP-deficiency significantly reduced serum creatinine and BUN levels compared to wild-type mice. Histological scores also showed decreased renal tubule dilation, tubular cell death, and cast formation in CHOP-deficient mice. This effect may be mediated by the regulation of pro-apoptosis, inflammation, and ROS-related signals, including cyclo-oxygenase-2 (COX-2), Caspase-3, and Caspase-8 [66]. CHOP-deficiency also attenuates renal fibrosis in the UUO model through a reduction in inflammatory infiltration, collagen deposition, and interstitial fibrotic area, and a repression of the expression of fibrotic markers including fibronectin, collagen, and α-SMA. The underlying mechanism may be mediated through the attenuation of CHOP-mediated inflammatory factors including IL-1β and TGF-β1 production, as well as PI3K/Akt activity [125]. These findings are consistent with the liver fibrotic murine model, in which CHOP deficiency attenuates hepatic fibrosis, inflammatory gene expression, and oncogenesis [126].

In terms of therapeutic approaches, one notable study on malignant cells states that the PERK-CHOP pro-apoptotic pathway is the barrier to malignancy. At the same time, the deletion of CHOP increases tumor incidence, and the molecular chaperone p58^IPK^ selectively attenuates PERK-CHOP-mediated apoptosis [127]. Since the repression of CHOP expression leads to some beneficial effects, the modulation of CHOP expression might offer potential in dealing with ER stress-mediated kidney injury. In contrast, CHOP deficiency results in more severe kidney injury in the LPS-induced acute kidney injury model [128]. LPS injection induces the activation of UPRs, including XBP1s, GRP78, GRP94, and CHOP. However, CHOP deficiency leads to higher BUN levels, albuminuria, and apoptotic cell death compared to WT mice under LPS treatment. The authors suggest that the mechanism is independent of the modulation of UPR singling; instead, it is mainly caused by changes in the inflammatory response of the downstream target C/EBP family proteins. CHOP might play an anti-inflammation role in LPS-induced kidney injury. Additionally, we should carefully discuss the role of CHOP in various kidney diseases, especially in kidney damage that is mainly caused by the inflammatory response.

## 8. Other Beneficial Effects in Moderating the ER Protein Homeostasis

### 8.1. Pre-Conditioning ER Stress

ER stress pre-conditioning showed protective effects in many studies, both in vitro and in vivo. In 1999, Bush and colleagues revealed that ER stress pre-conditioning protects ATP depletion-induced cell damage [129], and that pretreatment with tunicamycin could protect mice from acute ischemic injury [130]. This protective effect could be seen in other in vitro disease models, such as prolonged ATP depletion or oxidative stress-induced cardiomyocytes injury [131]. The underlining mechanisms may involve the prevention of increased intracellular Ca^2+^ concentration and ERK activation, and the decreasing of JNK activation [132], or they may be caused by the preserving of intercellular junctions, cytoarchitecture, and cell-substratum interactions in ATP-depleted epithelial cells. In addition, the administration of the ER stress inducers, tunicamycin or thapsigargin, significantly reduced mesangial proliferation and the adhesion of Bowman’s capsule to the glomerular tuft and proteinuria in a rat glomerulonephritis model [62].

### 8.2. AMPK Activation

AMP-activated protein kinase (AMPK) is a serine/threonine kinase that is thought to be a metabolism modulator that controls cellular and whole-body energy balance [133]. Recent studies showed that AMPK activation might protect renal cells from ER stress-induced injury. Administration of metformin, an AMPK activator, protects renal tubular cells against albumin loading-induced ER stress via inhibition of ROS through the induction of thioredoxin, an endogenous antioxidative molecule, and the suppression of BiP expression in an albumin-overloaded rat model [134]. Furthermore, AMPK suppressed tunicamycin, thapsigargin and/or TGF-β, angiotensin II, aldosterone, and high-glucose-induced ER stress in tubular epithelial cells [135]. Metformin also suppressed ER stress and fibrosis in both tunicamycin-induced AKI and UUO mouse models [135].

### 8.3. Roles of Chemical Chaperone 4-PBA and TUDCA

Both 4-phenylbutyrate (4-PBA) and TUDCA are small molecular weight chemical chaperones. The FDA approved 4-PBA for children with urea cycle disorders. They were also shown to have therapeutic potential for neurodegenerative diseases [136] and several types of cancers [137,138,139]. Both compounds assist in the adequate folding of protein in order to relieve ER stress-associated diseases [69,140,141], including renal fibrosis and diabetic nephropathy. In STZ-induced diabetes nephropathy, 4-PBA and TUDCA reduced the excretion of albuminuria and the expression levels of BiP, ATF6, PERK, JNK, and CHOP, and the inflammatory mediators. The chemical chaperons also restored the adaptive UPRs molecule XBP1s [52,87]. Furthermore, the chemical chaperones exerted an anti-fibrotic effect in post-injured UIRI or UUO mice models. Notably, recent research found that ER stress is involved in angiotensin II-induced NLRP3 inflammasome activation, and pretreatment with 4-PBA could reduce the expression of both NLRP3 and inflammatory cytokines, suggesting that 4-PBA serves as a potential agent to reduce renal fibrosis [142] (Table 1).

**Table 1:** Summarized the therapeutic effect of UPR modulators.

Chemical	Mechanism of Action	Animal Model	Therapeutic Effect	Ref.
4-PBA	unfolded protein folding	UIRI, UUO, Dahl salt-sensitive rat, STZ-DN	Yes	[57,69,140,141]
GSK2606414	PERK inhibitor	Prion infected mice	Not clear	[143]
Guanabenz	eIF2α phosphatase inhibitor	N/A	Not clear	[7,123]
KIRA6	IRE1 RNase inhibitor	Akita Mouse	Yes	[103]
Salubrinal	eIF2α phosphatase inhibitor	Cisplatin, Cadmium, Arsenic, Paraquat, Cyclosporine	Not clear	[64,120,121]
TUDCA	unfolded protein folding	UIRI, STZ-induced DN	Yes	[87,140]
HLJ2	XBP1s agonist	UC	Yes	[115,144]
(±)-8-ADC	XBP1s agonist	UC	Yes	[116]
Quercetin	IRE1 RNase activator	UUO, DN, Cadmium, ADAM	Yes	[104,105,106,107,108,109,110,111,112]

## 9. Conclusions

A number of therapeutic strategies related to UPR signaling were investigated in recent years; however, most focused on CNS disease, cancer, or diabetes [126,145]. In this article, we highlighted the findings of recent publications on the use of UPRs signals to target renal diseases or fibrosis progression either directly or indirectly, as well as some UPR modulators. We suggest that the modulation of phosphorylation-dependent PERK and IRE1 activities might be a promising direction in future pharmaceutical investigations [146]. As for the UPR modulator, there is lack of prominent reports, but based on the mechanisms of pathophysiology, it might still be a potential therapeutic target. Nevertheless, UPR modulators play the role of a double-edged sword, as they might induce either the systemic activation or inhibition of UPRs signaling, and be accompanied by unpredictable cellular stress. However, it is worth noting that the activation of adaptive UPR molecules, especially XBP1s in DN, podocyte-defective, or inflammatory models, is a promising therapeutic approach. Furthermore, the elimination of unfolded proteins or the enhancement of the ERAD function is also beneficial for kidney injury. In contrast, the role of the apoptotic UPR molecule CHOP is still not conclusive. For clinical applications of UPR modulators in renal fibrosis, some candidates, such as 4-PBA and TUDCA, are involved in ongoing DN clinical trials. Still, most of the compounds were only confirmed in vivo or in vitro. Therefore, more studies are required to confirm the beneficial effects of the modulation of UPRs on renal diseases.

## Data Availability

Not applicable.

## Abbreviations

4-PBA	4-phenylbutyric acid
ACEI	Angiotensin-converting enzyme inhibitor
AKI	Acute kidney injury
AMPK	AMP-activated protein kinase
ANG	Angiogenin
ATF4	Activating transcription factor 4
ATF6	Activating transcription factor 6
BiP	Binding immunoglobulin protein
CHOP	CAAT/enhancer-binding protein (C/EBP) homologous protein
CKD	Chronic kidney disease
COX-2	Cyclo-oxygenase-2
CREB	cAMP response element-binding protein
DN	Diabetic nephropathy
EDEM	ER degradation-enhancing alpha-mannosidase-like protein
eIF2α	Eukaryotic translation initiation factor 2α
EMT	Epithelial-mesenchymal transition
ER	Endoplasmic reticulum
ERAD	ER-associated protein degradation
FIP	Familial interstitial pneumonia
GADD34	Growth arrest and DNA-damage-inducible 34
GRP78	Glucose-regulated protein 78
HDAC3	Histone deacetylase 3
IBD	Inflammatory bowel disease
IRE1α	Inositol-requiring enzyme 1 α
JNK	c-Jun amino-terminal kinases
KIRA	Kinase-Inhibiting RNase Attenuators
NLRP3	NOD-, LRR- and pyrin domain-containing protein 3
PDI	Protein disulfide isomerase
PP1	Phosphatase-1
RIDD	Regulated IRE1-dependent decay
ROS	Reactive oxygen species
SPC	Surfactant protein C
STZ	Streptozotocin
TNRF	Tumor necrosis factor receptor
TRAF2	Tumor necrosis factor receptor-associated factor 2
TUDCA	Tauroursodeoxycholic acid
UC	Ulcerative colitis
UPR	Unfolded protein response
UUO	Unilateral ureteral obstruction
XBP1	X-box binding protein 1
XBP1s	Spliced isoform of XBP1
XBP1u	Unspliced isoform of XBP1
